# Cross-cultural adaptation of the Post-Intensive Care Syndrome Questionnaire to the Brazilian population: preliminary work

**DOI:** 10.62675/2965-2774.20250104

**Published:** 2025-09-09

**Authors:** Guilherme de Oliveira Rodrigues, Marcelo Velloso, Arnaldo Santos Leite, Alexandre Guimarães de Almeida Barros, Isadora Alves Ventura Marciano, Ingrid de Castro Faria, Lucas de Oliveira Cândido, Carolina Coimbra Marinho

**Affiliations:** 1 Universidade Federal de Minas Gerais Posgraduate Program in Rehabilitation Sciences Belo Horizonte MG Brazil Posgraduate Program in Rehabilitation Sciences, Universidade Federal de Minas Gerais - Belo Horizonte (MG), Brazil.; 2 Universidade Federal de Minas Gerais Department of Physiotherapy Belo Horizonte MG Brazil Department of Physiotherapy, Universidade Federal de Minas Gerais - Belo Horizonte (MG), Brazil.; 3 Universidade Federal de Minas Gerais Faculty of Medicine Department of Clinical Medicine Belo Horizonte MG Brazil Department of Clinical Medicine, Faculty of Medicine, Universidade Federal de Minas Gerais - Belo Horizonte (MG), Brazil.; 4 Universidade Federal de Minas Gerais Faculty of Medicine Belo Horizonte MG Brazil Faculty of Medicine, Universidade Federal de Minas Gerais - Belo Horizonte (MG), Brazil.; 5 Universidade Federal de Minas Gerais Faculty of Physiotherapy Belo Horizonte MG Brazil Faculty of Physiotherapy, Universidade Federal de Minas Gerais - Belo Horizonte (MG), Brazil.

Survivors of critical illness may experience physical, cognitive, and mental impairments, a condition known as post-intensive care syndrome (PICS). Assessment, however, is challenging because the different domains need separate assessment tools.^(1,2)^

The Post-Intensive Care Syndrome Questionnaire (PICSQ) was specially developed to assess PICS. Initially in English, it contains 18 questions, 6 in each of the 3 domains, answered on a 4-point Likert scale: zero (never) to 3 (always). The highest score, 54, signifies the severity of PICS.^(3)^ We aimed to culturally adapt the questionnaire to the Brazilian population.

This cross-cultural adaptation was authorized by the original authors of the instrument, Professors Yeon Jin Jeong and Jiyeon Kang, and was approved by the Research Ethics Committee of the *Universidade Federal de Minas Gerais* (UFMG), under protocol number 4331597. All participants provided written informed consent.

The work was performed in stages:^(4-6)^ preparation, including literature review, assembling the team of translators and authorization from the original authors; translation, independently by two native speakers of Brazilian Portuguese fluent in English, one of them familiar with PICSQ; reconciliation and synthesis of the two versions, considering the semantic, idiomatic, cultural, and conceptual equivalence to the original PICSQ, and production of a synthesized version (T1-2) by the translators and a third researcher; back-translation of version T1-2, independently, by two native English speakers, fluent in Brazilian Portuguese and unfamiliar with the original PICSQ; review and harmonization of the two back-translated versions by a committee composed of two doctors from the research team and a linguistics specialist, creating a back-translated synthesized version (BT1-2); approval of the BT1-2 by the original authors; pre-test of T1-2 (pre-final version) to assess inter-rater reliability and agreement, performed with patients discharged from the *Hospital Universitário* of the UFMG, at Belo Horizonte (MG, Brazil).

The pre-final version (Table 1) was independently applied to each participant by two interviewers, a physiotherapist and a medical undergraduate student, via telephone call 1 month after hospital discharge, with an interval of 24 to 48 hours. Participants were asked about any difficulties understanding the questions. Interviews were conducted by telephone due to restrictions during the coronavirus disease (COVID-19) pandemic. Data were collected using the REDCap electronic capture tool^(7)^ and analysed with Statistical Package for the Social Sciences (SPSS), IBM, version 25. Participants were adults admitted to the ICU for at least 5 days or on mechanical ventilation for 72 hours, and who could be contacted by telephone after discharge. Internal consistency was assessed using Cronbach's alpha and considered acceptable (> 0.7) or high (> 0.90).^(8)^ Inter-observer agreement was assessed by the intraclass correlation coefficient (ICC) and considered excellent (≥ 0.90), good (≥ 0.75), moderate (0.5 - 0.75), or poor (< 0.5). The Tyson and Connell clinical utility scale^(9)^ was used to quantify the speed with which the instrument can be applied, ease of understanding, application, scoring, cost, need for specialized equipment, and portability.

**Table 1:** Post Intensive Care Syndrome Questionnaire - Translation and cultural adaptation to Brazilian Portuguese

		Nunca (+0)	Às vezes (+1)	Na maior parte do tempo (+2)	Sempre (+3)
**1**	*É difícil decorar números.*				
**2**	*As pessoas ao meu redor dizem que eu repito o que eu já disse antes.*				
**3**	*É difícil para mim encontrar o caminho.*				
**4**	*Não consigo me concentrar na leitura.*				
**5**	*Adminstrar dinheiro é difícil.*				
**6**	*Eu me confundo com a data ou hora.*				
**7**	*Minhas juntas estão rígidas.*				
**8**	*A pega da minha mão está fraca.*				
**9**	*Eu mal posso subir as escadas.*				
**10**	*Meu desempenho sexual se deteriorou.*				
**11**	*Eu me canso facilmente.*				
**12**	*Eu me sinto doente no meu corpo todo.*				
**13**	*Meu coração está abafado.*				
**14**	*Eu tenho pesadelos.*				
**15**	*Eu estou preocupado (a).*				
**16**	*Eu estou irritado (a) ou com raiva.*				
**17**	*Eu me assusto facilmente.*				
**18**	*Eu não tenho nenhuma esperança.*				
	**Escore total**				

**Figure 1:**
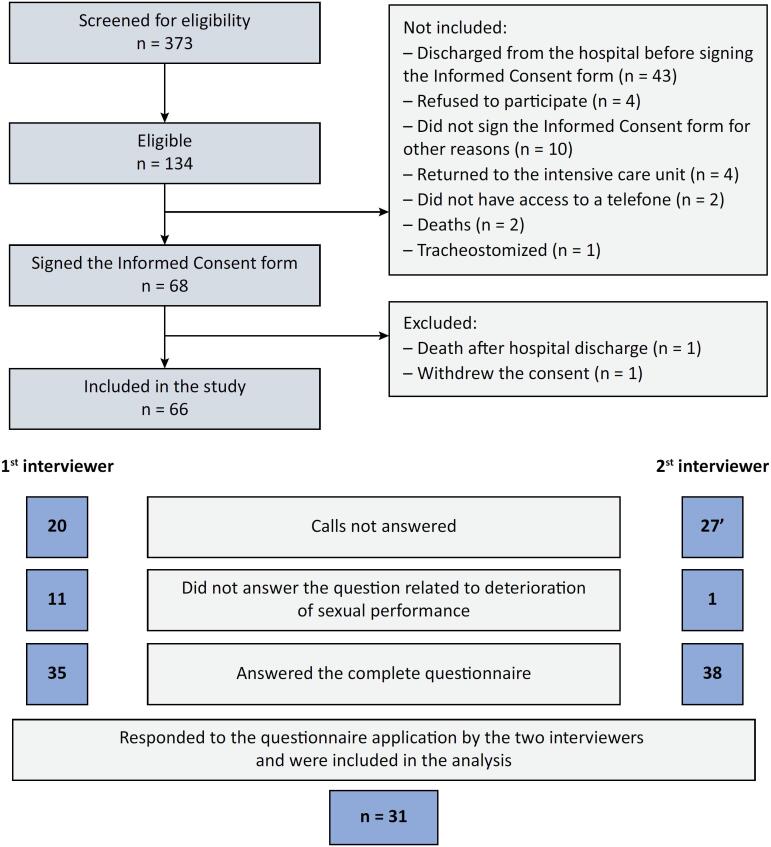
Flowchart of study recruitment.

Between October 30, 2020, and December 14, 2021, 373 individuals were assessed for eligibility, 134 were eligible, 66 were included (age 54.8 ± 15.6; 59.1% women, 59.1% nonsurgical), and 31 responded to the pre-final version by two interviewers (Figure 1). Internal consistency was acceptable for both interviewers (Cronbach's alpha 0.87 and 0.88). Inter-rater agreement was good (ICC 0.79; p = 0.0001). Time to complete was 4.6’± 1.7 and 5.0’ ± 2.35 for the first and second interviewers. Mean score was 14.26 ± 9.4 and 16.03 ± 10.2 (p = 0.132). Tyson and Connell clinical utility scale was 10 in 10.

The interviewees understood all items in the questionnaire, and no changes were necessary.

The pre-final version (Table 1) of the questionnaire is reliable and reproducible. We suggest that it be adopted as the final version and named PICSQ-Br.

The main limitation is that the work was performed in a single center, which may not represent regional differences in language. Clinimetric evaluation and validation are underway to assess applicability in the Brazilian population. The tool can be used to identify PICS in the post-acute phase and for sequential assessment in recovery, in addition to standardizing research in the field.
